# The effect of postmenopausal hormonal drop on optic nerve head and peripapillary perfusion using optical coherence tomography angiography (OCTA)

**DOI:** 10.1038/s41598-022-22844-3

**Published:** 2022-10-28

**Authors:** Mahmoud Fathy, Alia Noureldine, Hala M. Elmofty, Doaa Ahmad Tolba

**Affiliations:** Department of Ophthalmology, Faculty of Medicine, Kasr Al Ainy Hospital, Cairo University, Cairo, 11956 Egypt

**Keywords:** Diseases, Risk factors, Signs and symptoms, Optics and photonics

## Abstract

We studied the effect of menopause with subsequent estrogen drop on optic nerve head structure and peripapillary vasculature. This cross-sectional analytic study was carried out on 100 eyes of 100 patients; patients were divided into a premenopausal group (50 eyes) and a postmenopausal group (50 eyes). Optical coherence tomography was done to evaluate retinal nerve fiber layer thickness (RNFLT) and optical coherence tomography angiography (OCTA) to assess the peripapillary capillary vessel density. RNFLT as well as the peripapillary vessel density (VD) were significantly lower in the postmenopausal group (*P* value < 0.001) with increasing age, hormonal drop, and higher intraocular pressure (IOP), specifically in the inferior quadrant. However, the negative correlation between IOP and VD (r = − 0.541) was stronger than its negative correlation with RNFLT (r = − 0.318). Postmenopausal hormonal changes lead to a significant rise in IOP-although still not glaucomatous- and a decrease in the RNFLT and perfusion of the optic nerve. This confirms the relation between hormonal drop and glaucoma in postmenopausal women. Changes in peripapillary vascular density were more evident than RNFL in correlation with IOP and age changes. So, OCTA can be used to detect early optic nerve affection.

## Introduction

Despite the global increase in life expectancy, the age of menopause remains fixed. As the proportion of older women increases, an increasing prevalence of menopause and postmenopausal diseases may be expected to be seen^1^. Postmenopausal hormonal changes, especially the decline in estrogen level, are thought to play an important role in increasing the incidence of ocular symptoms and ocular diseases^2^.

Estrogen is a known vasodilator and neuroprotective hormone. Its postmenopausal withdrawal can affect the retina because of the presence of estrogen receptors in the retina and its vasculature^3^. In addition, it can increase the likelihood of glaucomatous optic nerve damage^4^, because of the effect of estrogen on lowering the intraocular pressure (IOP)^5^.

Currently, there is accumulating evidence that suggests a decrease in RNFLT in postmenopausal women^6^. However, no studies investigated the peripapillary vasculature in postmenopausal women to support similar changes. So, in this study, we investigated the effect of postmenopausal hormonal change on ONH and peripapillary perfusion.

## Materials and methods

This cross-sectional analytic study was carried out on 100 patients selected from the general Outpatient Clinics of Kasr Alainy school of medicine, Cairo University Hospitals. Informed consent was obtained from all participants prior to any study procedure.

### Patients’ selection

Premenopausal and postmenopausal healthy women aged 40–60 years with a best corrected visual acuity (BCVA) ≥ 0.8 were recruited. Pregnant and lactating women, or women who were currently or previously using oral contraceptives or hormone replacement therapy (within 6 months prior to enrollment) were excluded. We also excluded subjects with a history of systemic or local disease that could affect ONH or retinal perfusion, such as diabetes mellitus, hypertension, cardiac disease, glaucoma patients, or suspects (either open or closed angle), anemia, or any neurological disease. We also excluded candidates with a history of previous ocular surgery or refractive myopia greater than − 6 diopters or hyperopia greater than + 4 diopters. In addition, those with any media opacity not allowing good fundus visualization and OCTA imaging were excluded. Patients were divided into two groups: Group 1 (Premenopausal): 50 eyes of 50 patients while group 2 (Postmenopausal): 50 eyes of 50 patients. The eyes of all participants were evaluated for BCVA using Snellen’s chart (in decimals), slit-lamp examination, gonioscopy, IOP measurement using Goldman applanation tonometry, dilated fundus examination by binocular indirect slit-lamp biomicroscopy, detection of serum estrogen level (in the mid-follicular phase of the cycle in the premenopausal group) and finally SD-OCT and OCTA (Optovue Inc., Fremont, CA, USA), using split-spectrum amplitude-decorrelation algorithm. Each image set comprised two raster volumetric patterns (one with vertical priority and one with horizontal priority) that scanned an area of 4.5 × 4.5 mm centered on ONH to assess RNFLT and VD in the peripapillary area. Each volume comprised 216 line-scan locations at which five consecutive B-scans were obtained. Each B-scan contained 216 A-scans, which compared the consecutive B-scans at the same location to detect flow based on motion contrast. An En-face angiogram was obtained using the maximum flow (decorrelation value) projection. The OCTA images were co-registered with OCT B-scans that were obtained concurrently to enable visualization of both the vasculature and structure in tandem.

### Analysis of OCT A and OCTA Images

The built-in Angio Analytics software was used to evaluate VD and RNFLT. The software defines the peripapillary region as a 1.0 mm wide round annulus extending from the optic disc boundary. The disc margin is automatically detected based on Bruch’s Membrane Opening (BMO), and both cup and rim are measured within the BMO plane. The ONH map protocol was used to obtain RNFLT measurements which were calculated in a 10 pixel-wide band along a circle of 3.45 mm in diameter centered on the ONH. The overall average RNFLT as well as that in the superior, inferior, nasal, and temporal quadrants were used. Peripapillary VD was defined as the percentage of the area occupied by the vessels in the peripapillary region. The software calculated the whole image vessel density (wiVD) in the entire 4.5 × 4.5 mm^2^ image. Overall peripapillary VD, as well as the VD measurements of the 4 quadrants, were calculated. Color maps were also used to show the VD. All OCTA scans were evaluated for image quality and segmentation errors. Quality index lower than 6 or images with persistent motion artifacts as doubled vessel pictures and artifact lines were excluded. If both eyes were eligible, a random blinded selection was done. In case of high refractive error or media opacity hindering the OCT scanning (e.g., dense cataract, nebulous cornea), the other eye was chosen.

### Statistical analysis

Data was coded and entered using the statistical package for the Social Sciences (SPSS) version 26 (IBM Corp., Armonk, NY, USA). Data was summarized using mean and standard deviation. Comparisons between groups were done using the unpaired t test^7^*.* Correlations between quantitative variables were done using the Pearson correlation coefficient. Multivariate stepwise linear regression analysis was done to detect if age, estrogen and IOP act as independent predictors of densities^8^*.* *P*-value less than 0.05 was considered statistically significant*.*

### Ethical approval

This report was approved by the ethical committee of at Cairo University and followed the tenets of the Declaration of Helsinki.

### Consent to participate

All participants received oral and written consents as mentioned in methodology.

## Results

### Demographic and clinical characteristics

One hundred eyes of one hundred patients were enrolled in the study divided into fifty eyes into each of the premenopausal and postmenopausal groups. The Patients’ age ranged from 41 to 48 years with a mean age of 43.22 ± 2.18 years in the premenopausal group and from 52 to 60 years with a mean age of 54.56 ± 3.80 years in the postmenopausal group. The estrogen level was significantly lower in the postmenopausal group. Postmenopausal women also had significantly lower BCVA and higher IOP than the premenopausal ones (*P* value < 0.001). Table 1 summarizes the clinical characteristics of the study population.

**Table 1:** Demographic and clinical characteristics of the study population.

	Premenopausal group		Postmenopausal group		P value
	Mean	Standard deviation	Mean	Standard deviation	
Age (Years)	43.22	2.18	54.56	3.80	< 0.001
BCVA (Decimals)	0.94	0.06	0.81	0.07	< 0.001
CCT (um)	577.92	27.22	506.46	14.27	< 0.001
SE (diopters)	0.21	1.05	− 0.01	1.54	0.203
IOP (mmHg)	13.54	1.18	18.18	1.66	< 0.001
VCDR	0.31	0.11	0.32	0.09	0.351
Estrogen (pg/ml)	232.98	8.30	17.24	2.99	< 0.001

*BCVA* Best corrected visual acuity, *CCT* central corneal thickness, *SE* spherical equivalent, *IOP* Intraocular pressure, *VCDR* vertical cup to disc ratio.

### Retinal nerve fiber layer thickness

**Figure 1:**
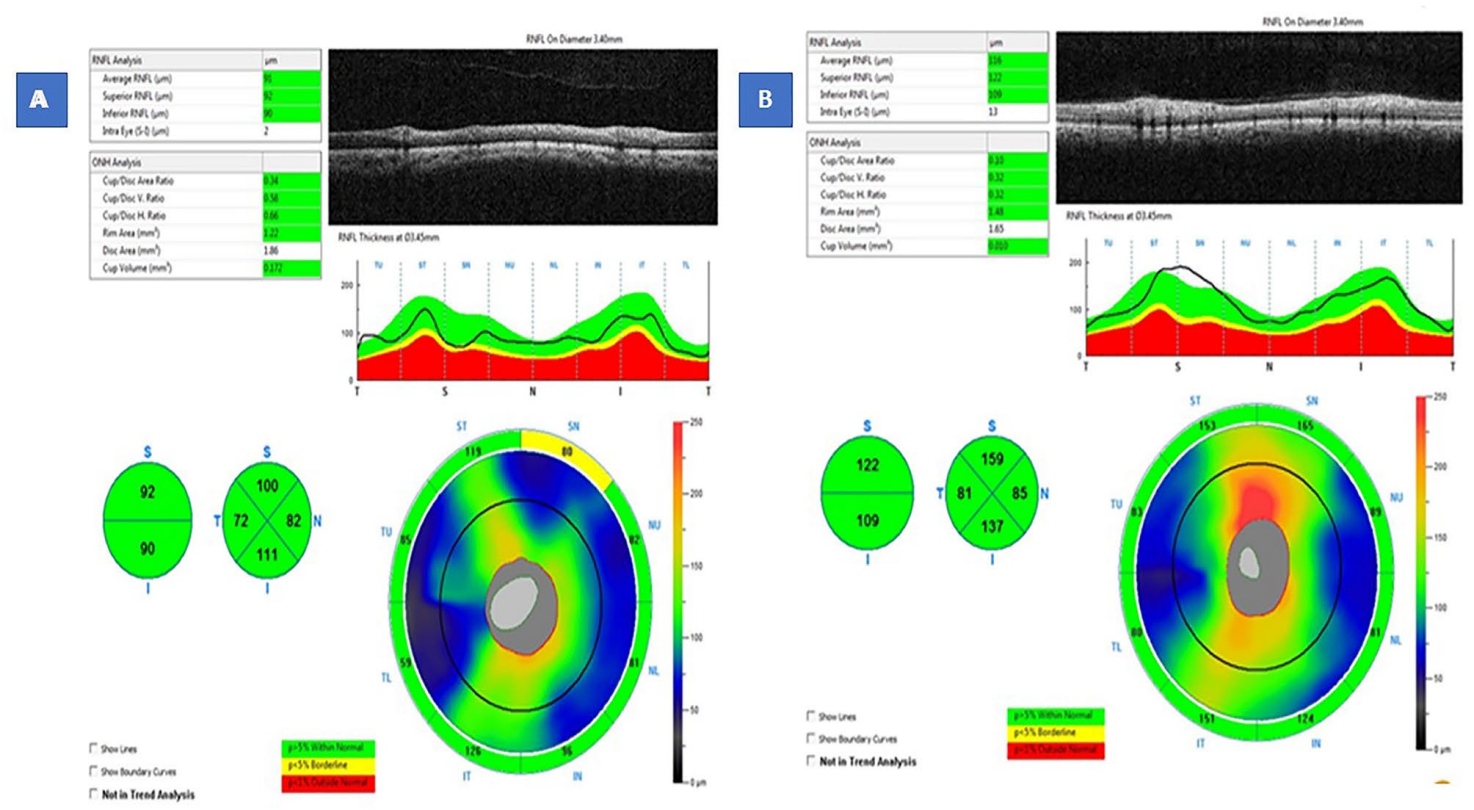
Example of RNFLT maps in a postmenopausal and a premenopausal participant.

The RNFLT was analyzed in the four quadrants (superior, inferior, nasal, and temporal). The mean RNFLT was significantly thinner in the postmenopausal women (Fig. 1A) in all quadrants compared to the premenopausal ones (Fig. 1B) (*P* value < 0.001) as summarized in Table 2.

**Table 2:** Comparison between C in both groups.

	Premenopausal group		Postmenopausal group		*P* value
	Mean	Standard deviation	Mean	Standard deviation	
Superior NFLT (um)	128.28	12.90	113.58	5.20	< 0.001
Inferior NFLT (um)	129.12	13.21	110.78	11.85	< 0.001
Nasal NFLT (um)	85.66	7.08	73.22	9.49	< 0.001
Temporal NFLT (um)	76.58	7.42	66.44	7.80	< 0.001

*RNFLT* Retinal nerve fiber layer thickness.

### Peripapillary vessel density (VD)

**Figure 2:**
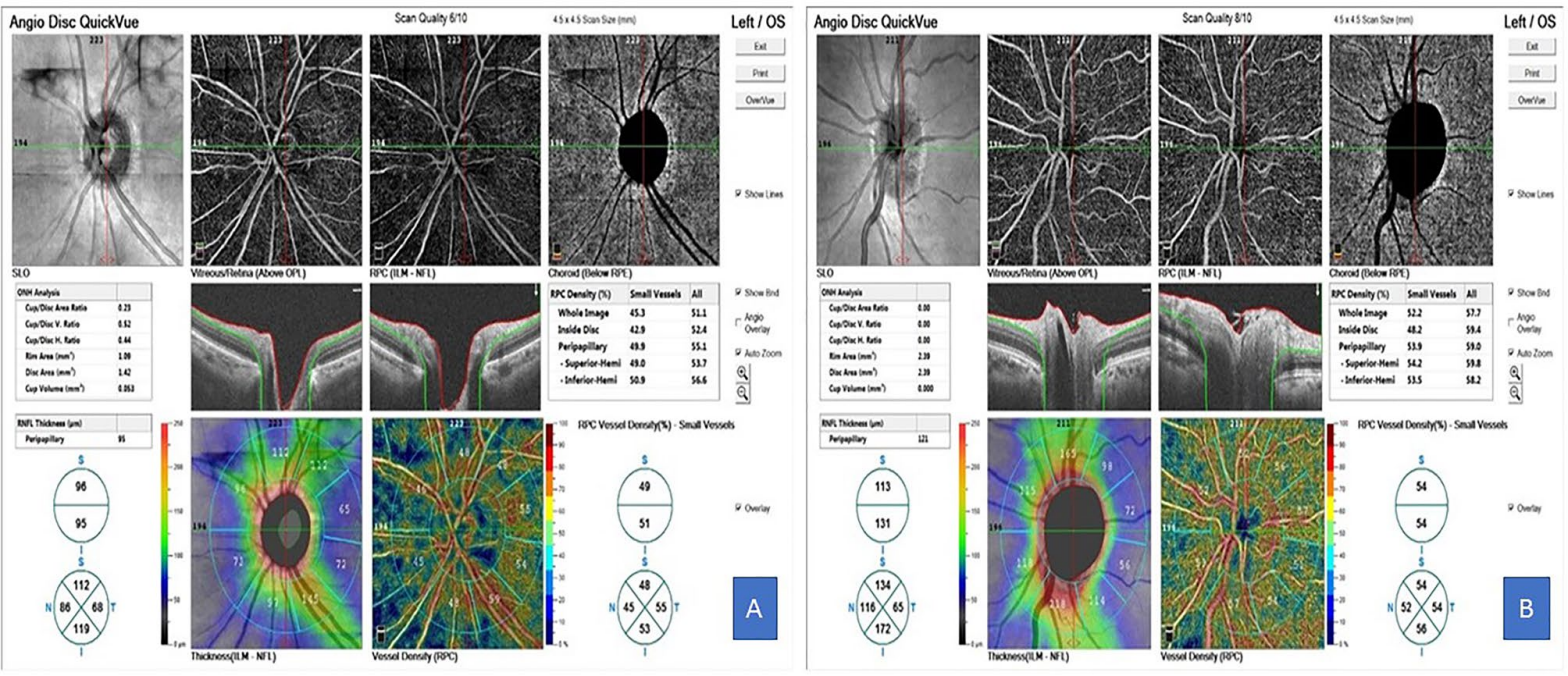
Example of a peripapillary OCTA scan 4.5 × 4.5 mm of ONH at Vitreous/Retinal level, RPC level, and Choroidal level, RPC density and thickness in a postmenopausal and a premenopausal participant.

The average of all peripapillary VD measurements was significantly lower in the postmenopausal group (Fig. 2A) (wiVD, inside disc VD, peripapillary VD, superior and inferior hemi) and in the 4 quadrants (superior, inferior, nasal, and temporal) compared to the premenopausal group (Fig. 2B) (*P* value < 0.001) as summarized in Table 3.

**Table 3:** Comparison between vessel denisty in both groups.

	Premenopausal group		Postmenopausal group		*P *value
VD (%)	Mean	Standard deviation	Mean	Standard deviation	
Wi VD (%)	51.55	1.98	48.80	2.72	< 0.001
Inside disc VD (%)	50.88	3.44	46.10	4.77	< 0.001
Peripapillary VD (%)	53.74	2.62	50.36	2.58	< 0.001
Superior hemi VD (%)	53.52	2.85	50.48	3.62	< 0.001
Inferior hemi VD (%)	53.84	2.86	51.21	2.26	< 0.001
Superior quadrant VD (%)	54.02	3.42	50.38	4.57	< 0.001
Inferior quadrant VD (%)	56.12	3.71	52.48	3.52	< 0.001
Nasal quadrant VD (%)	52.14	3.76	48.26	4.21	< 0.001
Temporal quadrant VD (%)	55.14	2.44	51.34	3.19	< 0.001

*VD* Vessel density, *Wi* Whole image.

Pearson correlation coefficients of peripapillary VD with RNFLT showed positive correlations in the vascular and structural changes. The correlation was statistically significant between RNFLT and VD in the inferior (*r* = 0.516, *P* < 0.001), nasal (*r* = 0.446, *P* = 0.001) and temporal quadrants (*r* = 0.577, *P* < 0.001) as shown in Table 4.

**Table 4:** Correlation between RNFLT and VD in the postmenopausal group.

Postmenopausal group		Superior RNFLT	Inferior RNFLT	Nasal RNFLT	Temporal RNFLT
Wi VD (%)	*r*	0.031	0.302	0.425	0.495
	*P* value	0.832	**0.033**	**0.002**	**< 0.001**
Inside disc VD (%)	*r*	-0.257-	-0.038-	0.227	0.240
	*P* value	0.072	0.796	0.114	0.094
Peripapillary VD (%)	*r*	0.016	0.094	0.516	0.510
	*P* value	0.912	0.515	** < 0.001**	** < 0.001**
Superior quadrant VD (%)	*r*	-0.099-	0.006	0.470	0.537
	*P* value	0.495	0.967	**0.001**	** < 0.001**
Inferior quadrant VD (%)	*r*	0.259	0.516	0.038	0.104
	*P* value	0.070	** < 0.001**	0.792	0.474
Nasal quadrant VD (%)	*r*	0.018	0.035	0.446	0.361
	*P* value	0.901	0.807	**0.001**	**0.010**
Temporal quadrant VD (%)	*r*	-0.097-	0.168	0.426	0.577
	*P* value	0.502	0.242	**0.002**	** < 0.001**

Significant values are in [bold].

*RNFLT* Retinal nerve fiber layer thickness, *Wi VD* Whole image vessel density, *VD* Vessel density, *r* Linear correlation coefficient.

### Correlation between IOP, estrogen and RNFLT and VD

**Figure 3:**
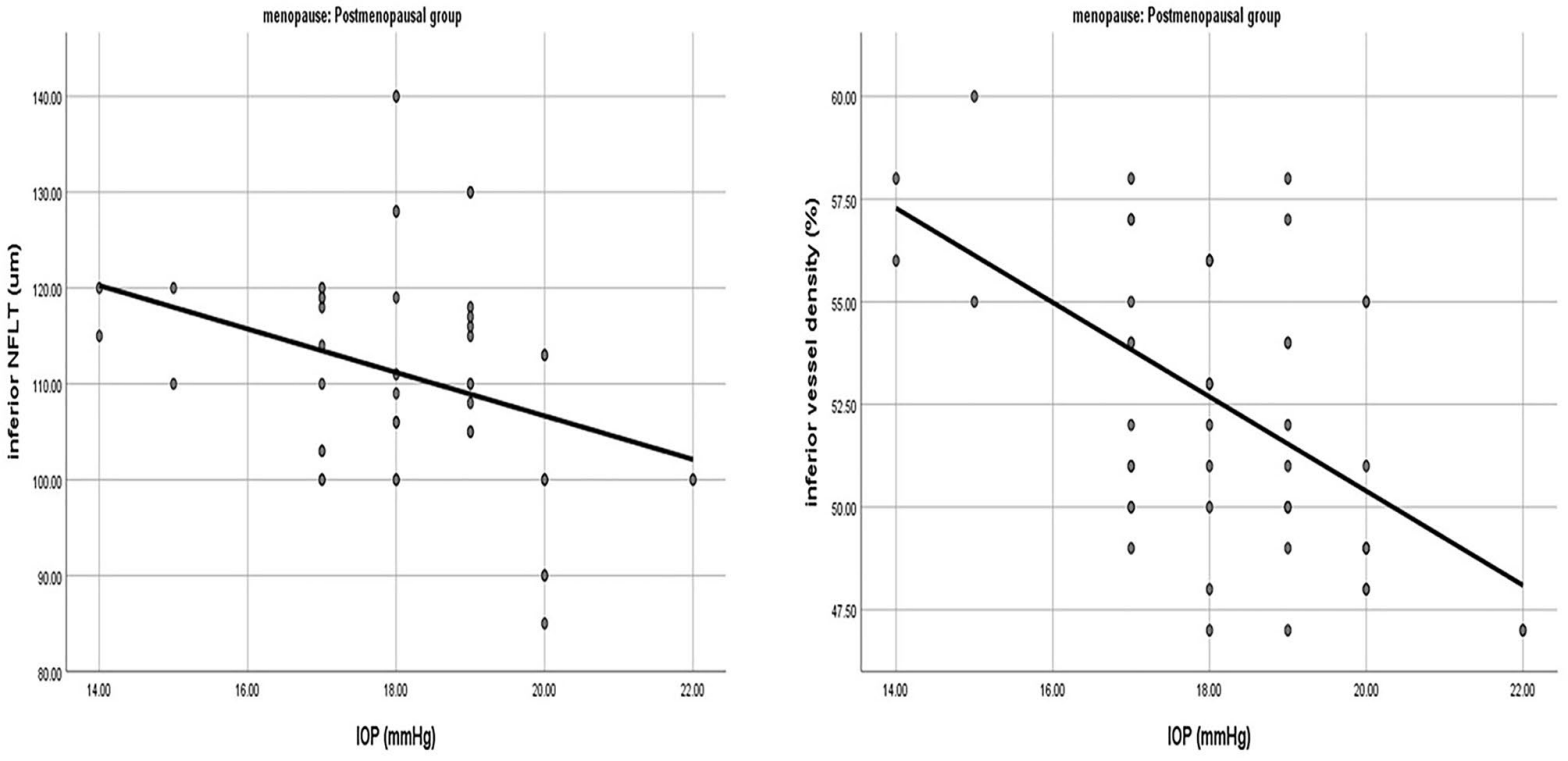
Correlation of IOP with inferior RNFLT and inferior VD in the postmenopausal group.

We studied the relation of IOP and estrogen with all parameters in the post-menopausal group. Analysis of Pearson correlation coefficients of the RNFLT with IOP showed a statistically significant negative correlation between IOP and RNFLT in the inferior quadrant only (r = − 0.318, *P* value 0.024) (Fig. 3).

Similarly, Pearson correlation coefficients of the VD with IOP revealed a significant negative correlation between IOP and VD in the inferior quadrant (r = − 0.541, *P* value < 0.001) (Fig. 3).

Analysis of the relation between the estrogen level and RNFLT and VD showed that there was a statistically significant positive correlation with nasal (r = 0.286, *P* value 0.044) and temporal RNFLT (r = 0.287, *P* value 0.043) and with peripapillary VD (r = 0.257, *P* value 0.029) and superior VD (r = 0.340, *P* value 0.016).

Multivariate stepwise linear regression analysis was done, with *age, estrogen level and IOP* (as independent predictors) and *vessel density* (as dependent variable). Results in Table 5 showed that estrogen level is an independent predictor for whole image, peripapillary superior and temporal VD affection. IOP has been shown to be a predictor of inferior VD affection.

**Table 5:** Multivariate stepwise linear regression analysis with age, estrogen and IOP as independent predictors and vessel density as dependent variable.

Model	Unstandardized coefficients		Standardized coefficients	t	*P* value	95.0% Confidence interval for B	
	B	Std. error	Beta			Lower bound	Upper bound
**Whole image**							
Estrogen	0.013	0.002	0.508	5.832	< 0.001	0.008	0.017
**Inside the disc vessel density**							
Age	-0.445-	0.124	-0.603-	-3.579-	0.001	-0.692-	-0.198-
IOP	0.933	0.269	0.534	3.468	0.001	0.399	1.468
Estrogen	0.019	0.009	0.430	2.230	0.028	0.002	0.036
**Peripapillary vessel density**							
Estrogen	0.016	0.002	0.551	6.542	< 0.001	0.011	0.020
**Superior vessel density**							
Estrogen	0.017	0.004	0.423	4.620	< 0.001	0.010	0.025
**Inferior Vessel density**							
IOP	-0.758-	0.128	-0.514-	-5.934-	< 0.001	-1.012-	-0.505-
**Nasal vessel density**							
Age	-0.322-	0.061	-0.471-	-5.279-	< 0.001	-0.442-	-0.201-
**Temporal vessel density**							
Estrogen	0.018	0.003	0.562	6.720	< 0.001	0.012	0.023

*IOP* intraocular pressure.

## Discussion

Postmenopausal drop in estrogen level is thought to play an important role in increasing the incidence of ocular symptoms and ocular diseases^2^. In this study, we evaluated the structural and vascular changes of the ONH in postmenopausal women in comparison to premenopausal women.

We found that the difference in the mean IOP was statistically significant between both groups with higher IOP in the postmenopausal group. This agreed well with the results of a study conducted by Siuw et al.^9^ who found a significant higher IOP in the postmenopausal group, and estradiol was shown to be a protective factor in reducing IOP among these women*.*

Regarding the optic nerve changes, older studies support the hypothesis that estrogen deficiency is involved in the pathophysiology of optic nerve aging and glaucomatous neurodegeneration through several mechanisms^6,9^. In this study, we found that the difference in RNFLT in the 4 quadrants was statistically significant between pre and postmenopausal groups. And by correlating the IOP with RNFLT in the different quadrants in the postmenopausal group, we found a statistically significant negative correlation between IOP and RNFLT in the inferior quadrant. Deschênes et al. in 2010 found that the measures of ONH topography indicated a significantly thicker RNFL in menopausal women on Hormonal replacement therapy (HRT) compared to women who never used HRT^3^. Comparably, Açmaz et al.^6^ in 2014 evaluated the RNFLT and choroidal thickness in premenopausal and postmenopausal women where no significant difference was found between the postmenopausal study and control groups regarding all the peripapillary RNFLT thickness parameters*.*

We found that all the average measurements of VDs are significantly lower in the postmenopausal group with a negative correlation between IOP and inferior hemi, and inferior quadrant only. No previous studies reported using OCTA to evaluate the peripapillary vasculature to compare with. Yet, others used different methods to predict ocular blood flow. As an example, Centofanti et al. in 2002 studied the pulsatile ocular blood flow (POBF) and have revealed gender and hormonal status-related alterations in the choroidal circulation^10,11^.

We found a statistically positive correlation between the loss of VDs and RNFLT in 3 out of the 4 quadrants. This agreed with previous studies stating that the radial peripapillary capillary densities correlated significantly with the RNFL thickness^12,13^.

We then compared the changes of RNFLT and VD with IOP. We found that with hormonal drop, increasing age and higher IOP, there was a decline in both RNFLT and peripapillary VD, especially in the inferior quadrant; but VD showed more significant negative correlation than RNFLT with IOP. So, according to this study, we can consider the assessment of VD by OCTA to be more sensitive in the early detection of peripapillary changes if IOP rises with menopause. This agreed with Lee et al.^14^ who reported that the inferior VD showed better diagnostic ability than most of the other OCT measurements including peripapillary RNFLT and macular ganglion cell inner plexiform layer (GCIPL) thickness in glaucomatous highly myopic eyes.

To our knowledge, this is the first study to elaborate the peripapillary vascular changes in menopausal women and correlate it with RNFLT and IOP. The limitations of this study are the lack of a group diagnosed with glaucoma to compare the changes with them. Axial length measurement would have been of additional asset to exclude changes caused by over stretching; but that could be forgiven since we excluded women with high refractive errors from the start. Also, it would have been of great value if we could follow up the premenopausal women to detect the changes that occur to them after menopause; but this needs a longer study duration.

## Data Availability

The datasets used and/or analysed during the current study available from the corresponding author on reasonable request.
